# Arm and Hand Movement: Current Knowledge and Future Perspective

**DOI:** 10.3389/fneur.2015.00019

**Published:** 2015-02-06

**Authors:** Renée Morris, Ian Q. Whishaw

**Affiliations:** ^1^Department of Anatomy, Translational Neuroscience Facility, School of Medical Sciences, UNSW Australia, Sydney, NSW, Australia; ^2^Department of Neuroscience, Canadian Centre for Behavioural Neuroscience, University of Lethbridge, Lethbridge, AB, Canada

**Keywords:** reaching, grasping, arm and hand movement, neuromuscular dysfunction, reach-to-grasp, motor control of movement

Reaching with the arm and grasping with the hand and fingers is a complex behavior that appears *in utero*, is elaborated over the first few years of life, and serves useful everyday functions throughout the course of human life. Several neurological conditions can impair the ability to produce arm and hand movements and so greatly impact on the quality of life and well-being of the affected individuals. Given the fundamental role that arm and hand movements play in everyday life, deficits related to arm and hand function are one of the most debilitating motor conditions. Neurological conditions that can affect arm and hand movements include autism spectrum disorder, Parkinson’s and Huntington’s diseases, amyotrophic lateral sclerosis, cerebral palsy, and stroke-related motor cortex damage as well as spinal cord injury at cervical levels. While arm and hand movement has received considerable attention from both clinicians and researchers from diverse scientific backgrounds, there are a number of broad research questions that still need to be addressed in this research field. The present Research Topic is entirely devoted to arm and hand movement in health as well as in disease. It is a compilation of original research papers and reviews, clinical case studies, hypothesis and theory articles, opinions, commentaries, and methods articles that cover important aspects of the topic from different perspectives.

In this volume, de Bruin et al. (1) present data that describe how healthy adults use space while performing a visually guided grasping task. A model for understanding hand functioning in children with cerebral palsy is proposed by Arnould et al. (2) while Johansson et al. (3) explore the effect of timing training on upper limb movement in three children with diplegic cerebral palsy. Parma et al. (4) compare the kinematics of the reach-to grasp movement in patients with vascular and idiopathic Parkinson’s disease whereas Aluru et al. (5) evaluate the effect of auditory constraints on motor performance at different stages after a stroke. Lawrence et al. (6) measure dexterous manipulation in a cross-sectional study comparing gender, age, and absence and presence of disease. Kirsh et al. (7) provide evidence to support the view that neurons outside the primary motor cortex – such as those populating the pontomedullary reticular formation and the spinal cord-drive movement and muscle synergies that primary motor cortex neurons then break up to create individual wrist and finger movements. Sacrey et al. (8) summarize current knowledge related to reaching and grasping in autism spectrum disorder. On the other hand, Whitwell et al. (9) reinstate patient DF’s amazing ability to use information regarding form and orientation of objects to guide skilled reaching actions despite her visual agnosia. In an opinion article, Moore (10) argues that nerve transfer is increasingly popular and is becoming the best treatment strategy for most brachial plexus damage as well as for patients with spinal cord injury at cervical levels. Vicario (11) provides a personal commentary on a paper from Hayashi et al. (12) and, in a review article, Karl and Whishaw (13) summarize the evidence that show that reaching and grasping are from distinct neural and evolutionary origins. Irvine et al. (14) contribute a methods article that assesses the reliability of the Irvine, Beatties, and Bresnahan (IBB) forelimb recovery scale. Fouad et al. (15) demonstrate that continuous viral-mediated brain-derived neurotrophic factor (BDNF) over-expression promotes spasticity in rats with spinal cord hemisections at cervical levels. Alstermark and Pettersson (16) bring evidence to show that lesions to the corticospinal tract that spare the cortico-reticulospinal pathway in the rat have no deleterious effects on skilled reaching and grasping. Finally, Tosolini et al. (17) describe how targeting the full length of the motor endplate region in the mouse forelimb with Fluoro-Gold increases the uptake of this neuroanatomical retrograde tracer in corresponding motor neurons.

We are delighted to present “Arm and Hand Movement: Current Knowledge and Future Perspective” as a Research Topic in Frontiers in Neurology. We feel that this wide-ranging compilation of articles by leading experts in upperlimb/forelimb movement and working either in clinical or basic research settings has offered fresh perspectives on the topic. We are thankful for the support of all the scientists who have contributed to this Research Topic and have shared with us their expertise and point of views. Their contributions have deepened our appreciation of the challenge that restoring arm and hand function in different pathologies represents. We invite the readers to experience the diversity in methodological approaches and experimental designs that together have led to broaden our understanding of this particularly wide field of research.

## Conflict of Interest Statement

The authors declare that the research was conducted in the absence of any commercial or financial relationships that could be construed as a potential conflict of interest.
